# Enhancing genomic prediction in *Arabidopsis thaliana* with optimized SNP subset by leveraging gene ontology priors and bin-based combinatorial optimization

**DOI:** 10.3389/fbinf.2025.1607119

**Published:** 2025-06-18

**Authors:** Qingfang Ba, Heng Zhou, Zheming Yuan, Zhijun Dai

**Affiliations:** Hunan Engineering and Technology Research Center for Agricultural Big Data Analysis & Decision-making, Hunan Agricultural University, Changsha, China

**Keywords:** genomic selection/prediction, SNP, subset selection, gene ontology, biological priors, *Arabidopsis thaliana*

## Abstract

With the rapid development of high-density molecular marker chips and high-throughput sequencing technologies, genomic selection/prediction (GS/GP) has been widely applied in plant breeding. *Arabidopsis thaliana*, as a common model organism, provides important resources for dissecting genetic variation and evolutionary mechanisms of complex traits. Quantitative traits are typically influenced by multiple minor-effect genes, which are often functionally related and can be enriched within gene ontology (GO) pathways. However, optimizing marker subsets associated with these pathways to enhance GP performance remains challenging. In this study, we propose an improved GS framework called binGO-GS by integrating GO-based biological priors with a novel bin-based combinatorial SNP subset selection strategy. We evaluated the performance of binGO-GS on nine quantitative traits from two *A. thaliana* datasets, comprising nearly 1,000 samples and over 1.8 million SNPs. Compared with using either the full marker set or randomly selected markers with Genomic BLUP (GBLUP), binGO-GS achieved statistically significant improvements in prediction accuracy across all traits. Similar improvements were observed across six additional regression models when applying binGO-GS instead of the full marker set. Furthermore, the selected markers for identical or similar morphological traits exhibited consistent patterns in quantity and genomic distribution, supporting the polygenic model of complex quantitative traits driven by minor-effect genes. Taken together, binGO-GS offers a powerful and interpretable approach to enhance GS performance, providing a methodological reference for accelerating plant breeding and germplasm innovation.

## 1 Introduction

One of the primary goals of plant breeding is to increase crop yields and accelerate the development of elite varieties, which is essential to ensure food security in response to the projected global population of 9.5 billion by 2050 (He and Li, 2020; Qaim, 2020). However, breeding for optimal performance remains a formidable challenge due to climate variability, evolving pathogens, limited arable land, and other biotic and abiotic stresses (Bali and Singla, 2022). Genomic selection/prediction (GS/GP), which predicts the genetic potential of individuals using genome-wide marker information, has emerged as a powerful approach to address these challenges and is now widely adopted in plant and animal breeding programs (Meuwissen et al., 2001; Bernardo and Yu, 2007; Heffner et al., 2009; Crossa et al., 2017).

GS enables the prediction of complex quantitative traits by incorporating thousands of single nucleotide polymorphisms (SNPs) distributed across the genome, irrespective of their individual statistical significance (Meuwissen et al., 2001). The widespread adoption of GS has been further fueled by decreasing genotyping costs and the availability of high-density SNP arrays and whole-genome sequencing data (Bernardo and Yu, 2007; Heffner et al., 2009; Crossa et al., 2017). Alongside traditional regression methods, machine learning (ML) techniques have been increasingly employed to capture nonlinear and high-order interactions among genomic features (Wolpert and Macready, 1997; González-Recio et al., 2014; Ma et al., 2014; Abdollahi-Arpanahi et al., 2020). However, despite these advances, the accuracy of genomic prediction remains constrained by the inclusion of large numbers of non-causal markers, which introduce noise and reduce model efficiency.

To improve the biological relevance of marker subsets used in GS models, researchers have explored integrating prior biological knowledge such as gene ontology (GO), gene networks, and metabolic pathways into predictive models (Lango Allen et al., 2010; O'Roak et al., 2012; Lage et al., 2012; Maurano et al., 2012; Peñagaricano et al., 2013). GO, in particular, provides curated annotations of gene function and biological processes, and markers associated with specific GO terms are more likely to be enriched for causal variants. For genomic prediction of photosynthesis and plant growth traits in *A. thaliana*, GO-informed features were incorporated into GBLUP models, but required extensive model fitting and faced challenges with marker redundancy (Farooq et al., 2021).

Directly including all SNPs linked to effective GO terms may introduce a substantial amount of irrelevant of weak-effect markers, which can obscure true signals and reduce model performance. This highlights the critical need for effective marker subset selection. Identifying an optimal SNP subset that balances strong-effect and weak-effect markers is crucial for accurate GS, especially under the polygenic nature of most complex traits. However, this task is computationally intractable (NP-hard), and heuristic or biologically guided methods are needed to navigate the search space efficiently. Our research group has previously proposed a series of feature selection approaches for trait prediction or pattern recognition problems (Ji et al., 2025; Qin et al., 2022; Dai et al., 2021; Dai et al., 2020; Dai et al., 2014), and similar efforts have been reported in GS studies using various selection and dimensionality reduction techniques (Xu et al., 2025; Yoshida and Yáñez, 2022; Piles et al., 2021; Luo et al., 2021).

In this study, we propose a novel GO-guided marker subset selection method called binGO-GS. The key idea is to leverage prior biological knowledge from GO annotations and combine it with a bin-based combinatorial selection strategy. We first select GO terms that are mapped with a sufficient number of SNPs and then apply a Monte Carlo strategy to estimate the optimal marker subset size. The SNPs are stratified based on *p*-values of genome-wide association study (GWAS) and iteratively combined to form the final subset using a heuristic bin-based optimization process. This approach captures both strong-effect and weak-effect markers while significantly reducing computational complexity. We validated the effectiveness of binGO-GS on two *Arabidopsis thaliana* datasets across nine quantitative traits and demonstrated its superiority over the full marker set and randomly selected markers across seven regression models, including GBLUP, RKHS (Gianola and Van Kaam, 2008), four Bayesian methods (Bayes A/B/C/LASSO) (Pérez and de Los Campos, 2014), and a deep learning model, DNNGP (Wang et al., 2023). The selected marker subsets also revealed trait-specific patterns consistent with the polygenic model, supporting the utility of biologically informed feature selection in GS applications.

## 2 Materials and methods

### 2.1 The *A. thaliana* datasets and quality control

The genotype data were retrieved from the genomes of 1,135 naturally inbred lines of *Arabidopsis thaliana* (Alonso-Blanco et al., 2016) and the GMI-MPI project of Arabidopsis 1,001 Genomes Project (https://1001genomes.org/data/GMI-MPI/releases/v3.1/), which includes high-quality resequencing data collected from across Eurasia, North Africa, and North America. These accessions represent the native distribution of *A. thaliana* and capture its global polymorphism landscape. The initial dataset contained 10,709,949 SNPs.

Two phenotypic datasets associated with the 1,001 Arabidopsis Genomes Project were used in this study. The first dataset includes 944 samples and five quantitative traits: two morphological traits (stem branching number (CL) and rosette leaf number (RL)) and three flowering time traits (days to 1 cm inflorescence stem elongation (DTF2), days to first flower opening (DTF3), and days from sowing to visible floral buds at the rosette center (DTF1)) (Grimm et al., 2017). The second dataset comprises 407 samples with four quantitative traits: one yield trait (rosette dry mass at fruit maturity, DM), two stem growth traits (scaling exponent, SE, and mean growth rate, GR), and one fruit development trait (fruit number at maturity, FN) (Vasseur et al., 2018). For clarity, these datasets hereafter referred to Arabi944 and Arabi407, respectively, based on their sample sizes.

Genotype data were subjected to the following quality control (QC) procedures using PLINK 1.9 (Purcell et al., 2007): SNPs with minor allele frequency (MAF) < 0.01 were removed; all markers passed Hardy‒Weinberg equilibrium filtering; linkage disequilibrium (LD) pruning was performed using a sliding window of 50 SNPs, a step size of 5, and an *r* ^2^ threshold of 0.95. After QC, 2,053,821 and 1,882,667 SNPs were retained for the Arabi944 and Arabi407 datasets, respectively. All subsequent analyses were based on these filtered markers. Samples with missing phenotypes were excluded on a per-trait basis. In the Arabi944 dataset, the number of retained samples per trait was 904 (CL), 850 (RL), 931 (DTF1), 923 (DTF2), and 936 (DTF3). In the Arabi407 dataset, the number of valid samples was 407 for DM, SE, and GR, and 396 for FN (Table 1).

**TABLE 1:** Summary of phenotypes, genotypes, and estimated genomic heritability in *Arabidopsis thaliana* datasets.

Dataset (No. SNPs)	Trait	No. Samples	*h* ^2^
Arabi944 (2053821)	CL	904	0.6715
	RL	850	0.7774
	DTF2	931	0.8915
	DTF3	923	0.8544
	DTF1	936	0.8812
Arabi407 (1882667)	DM	407	0.7976
	SE	407	0.7603
	GR	407	0.7296
	FN	396	0.6746

### 2.2 Genomic heritability estimation

Genomic heritability for each quantitative trait was estimated using GCTA (version: 1.93.3beta2) (Yang et al., 2011), providing a basis for comparing the predictive performance across traits (Table 1). The estimation involved two steps: first, a genomic relationship matrix (GRM) was constructed based on SNP markers; second, a linear mixed model was fitted using restricted maximum likelihood (REML) (Kenward and Roger, 1997) to partition phenotypic variance into genomic and residual components. Genomic heritability was then calculated as the proportion of total phenotypic variance explained by the genomic component.

### 2.3 Development of the binGO-GS pipeline

#### 2.3.1 Initial screening of marker subsets based on GO biological priors

The phenotypic variation of complex quantitative traits is typically influenced by intricate regulatory networks involving multiple genes. The Gene Ontology (GO) database, which provides a structured representation of gene function across biological processes, molecular functions, and cellular components, offers a valuable biological prior for identifying SNP markers related to gene regulatory networks. Specifically, each GO term consists of a group of genes associated with that term or its subordinate terms.

In this study, we first mapped GO terms to genes using the “org.At.tairGO2ALLTAIRS” function from the R package “org.At.tair.db”, which retrieves comprehensive and propagated gene sets for each GO term, including all subordinate terms. SNPs were then linked to these GO-annotated genes based on genomic positions, thereby establishing the association between GO terms and SNP markers (Farooq et al., 2021).

Through this procedure, we identified 7,645 GO terms, each associated with at least one SNP marker. To refine the candidate marker set, SNPs linked to genes with GO terms unrelated to the traits of interest (e.g., basic metabolic processes or stress responses) were excluded. Priority was given to SNPs annotated under GO terms biologically relevant to the target traits (e.g., photosynthesis, flowering regulation). Additionally, only GO terms whose associated gene regions collectively contained more than 200 SNPs were retained, ensuring adequate marker representation per GO term. As a result, 4,645 GO terms met the selection criteria. The SNP markers corresponding to these terms were merged and deduplicated, yielding 716,860 SNPs for the Arabi944 dataset and 660,238 SNPs for the Arabi407 dataset.

#### 2.3.2 Determining the upper limit of marker subset size

Following the initial GO-based screening, the spatial distribution of SNP markers across the genome was altered, potentially introducing local marker redundancy due to linkage disequilibrium (LD). To address this, we performed LD pruning using PLINK (Purcell et al., 2007) to reduce redundant information and ensure that the remaining markers were relatively independent.

Determining an appropriate upper limit for the number of SNP markers is critical for balancing prediction accuracy and model parsimony. While using all available markers can maximize information, it may also introduce noise and computational burden. Conversely, retaining too few markers risks omitting those linked to key QTLs. Prior studies have shown that randomly selecting a moderate number of markers (e.g., 100,000) evenly distributed across the genome does not lead to a significant decline in prediction accuracy. However, the optimal number of informative markers should ideally be determined in a data-driven manner.

**FIGURE 1:**
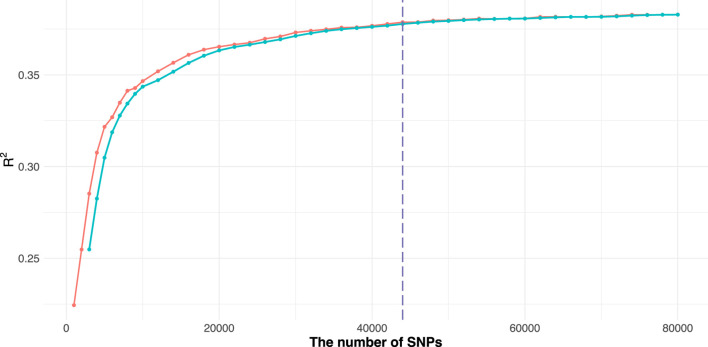
Determination of the optimal upper limit of marked subset size for the trait CL. The raw accuracy curve (red) represents the average prediction accuracy over 50 random samplings at each SNP subset size, while the smoothed curve (green) shows the moving average used to capture the overall trend. The dashed vertical line marks the point where the accuracy gain begins to plateau, indicating the selected upper limit of subset size.

In this study, we used a Monte Carlo approach to identify the upper limit of marker subset size. Specifically, we randomly sampled subsets of SNPs of increasing size, ranging from 1,000 to 80,000 (approximately 10% of the post-LD-pruned marker pool). For subset size under 10,000, the size was incremented by 1,000; for those above 10,000, increments of 2,000 were used. At each marker size, 50 random replicates were performed to evaluate prediction accuracy. We plotted the number of markers (X-axis) against the mean accuracy (Y-axis) across the 50 replicates (Figure 1).

To identify the point at which prediction accuracy plateaued, we implemented an automated inflection point detection strategy (Equations 1, 2). The curve was smoothed using a moving average over 3 or 5 sampling points:

$$MA\left(t\right)=\frac{y\left(t\right)+y\left(t-1\right)+y\left(t-2\right)}{3}$$ (1)

The slope of the smoothed curve was then calculated as:

$$Slope\left(t\right)=MA\left(t\right)-MA\left(t-1\right)$$ (2)

We considered the curve to have plateaued when the slope remained near zero or declined significantly over at least three consecutive intervals, or when a slope drop of over 20% relative to its prior average was observed. The corresponding marker quantity at the first point of this stable region was designated as the upper limit for subset size (Figure 1). Based on this procedure, the optimal marker subset sizes for the nine traits were determined as follows: CL, 58,000; RL, 54,000; DTF2, 54,000; DTF3, 54,000; DTF1, 50,000; DM, 56,000; SE, 46,000; GR, 44,000; FN, 66,000.

#### 2.3.3 Marker subset selection via bin-based combinatorial optimization

Incorporating SNPs identified through GWAS as fixed effects into genomic prediction models can significantly enhance prediction accuracy for quantitative traits (Zhang et al., 2014). To reduce spurious associations caused by population structure and relatedness, we performed GWAS by including three principal components (PCs) derived from the training set and a kinship matrix based on the full set of genome-wide markers as covariates in a linear mixed model (LMM). The analysis was implemented using GEMMA software (Zhou and Stephens, 2012).

For each SNP, a *p*-value was computed, and markers with *p* < 0.01 were retained. This threshold was selected based on empirical evaluation to balance signal strength and subset manageability. Using *p* < 0.05 included about 35,000 SNPs per trait on average, introducing excessive weak-effect markers that reduced complementarity with downstream bin-based optimization. Conversely, *p* < 0.005 typically retained fewer than 5,000 SNPs, necessitating more rounds of optimization to reach the desired subset size and increasing computational complexity. The threshold of 0.01 thus provided a practical compromise. These significant markers were further subjected to LD pruning to eliminate redundancy. The resulting marker set, enriched for SNPs likely to have strong associations with the target traits, was defined as Subset I.

**FIGURE 2:**
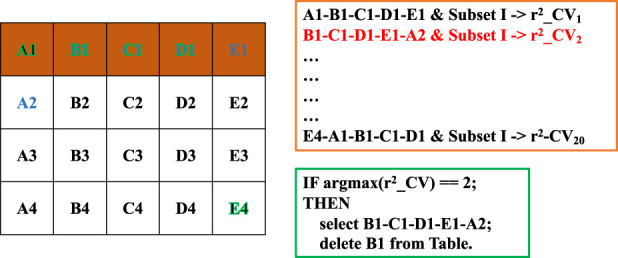
Illustration of the combinatorial optimization process using the chain-wise rolling strategy. Bins (A–E) represent different ranges of *p*-values, and each bin is further divided into four groups (1–4) based on ascending *p*-values (left table). A total of 20 SNP combinations are constructed by sequentially rotating the leading group across bins. For each combination, the SNPs are merged with those from Subset I, and cross-validation is performed to assess genomic prediction accuracy (orange frame). The combination yielding the highest accuracy is retained, and its leading group (e.g., B1) is excluded from subsequent selection rounds (green frame: pseudocode representation).

While using only highly significant markers can help reduce noise, it may omit many informative variants with moderate or small effects. Exhaustively searching for the optimal SNP combination from the entire genome is computational infeasible (NP-hard). Therefore, we developed a bin-based combinatorial optimization strategy, which integrates Subset I (strong-effect markers) and its complement set (containing potentially weak-effect markers). The procedure consists of the following steps:

1) Segmentation into bins and groups. The remaining SNPs (excluding Subset I) were first divided into 10 bins based on their GWAS *p*-values in ascending order: [0.01–0.1], [0.1–0.2], …, [0.9–1.0]. To further control subset size and ensure diversity within each bin, we grouped the SNPs in each bin into 10 subgroups using a sliding window of 0.01 on the *p*-values. Markers in lower-ranked groups of a bin could be complemented with markers from subsequent bins if necessary.
2) Chain-wise rolling combination search. To explore diverse combinations of weak-effect markers across bins without exhaustive testing (which would require evaluating 10^10^ combinations), we designed a multi-round chain-wise rolling strategy (Figure 2). Conceptually treating bins as experimental factors and groups within bins as their levels, we systematically rotated through the groups. For instance, in a scenario with 5 bins (A–E) and 4 groups per bin (1–4), the first combination would be A1-B1-C1-D1-E1, followed by B1-C1-D1-E1-A2, and so forth, until all group permutations were covered (e.g., E4-A1-B1-C1-D1). This strategy ensures that every group participates in at least one combination while maintaining good representation and orthogonality across bins.
3) Selection of optimal combinations per round. In each rolling round, the SNPs from one group combination were merged with Subset I, followed by LD pruning to remove redundant markers. The prediction accuracy was assessed via cross-validation on the training set. The combination yielding the highest accuracy was retained, and its associated markers were added to the final marker subset. The leading group of that combination was then excluded from further selection. The remaining 99 combinations proceeded to the next rolling round.
4) Subsequent rolling rounds. In subsequent rounds, it was no longer necessary to ensure that all bins were represented in every combination. Each new combination was merged with all previously selected markers (including Subset I), followed by LD pruning and cross-validation to identify the optimal combination for that round. This process was repeated iteratively until the total number of selected SNPs reached the upper limit of the marker subset size. After the final SNP set was determined, the complementary marker subset, i.e., all selected markers not present in Subset I, was designated as Subset II. This allowed us to evaluate the relative contribution of weak-effect versus strong-effect markers in genomic prediction.

**FIGURE 3:**
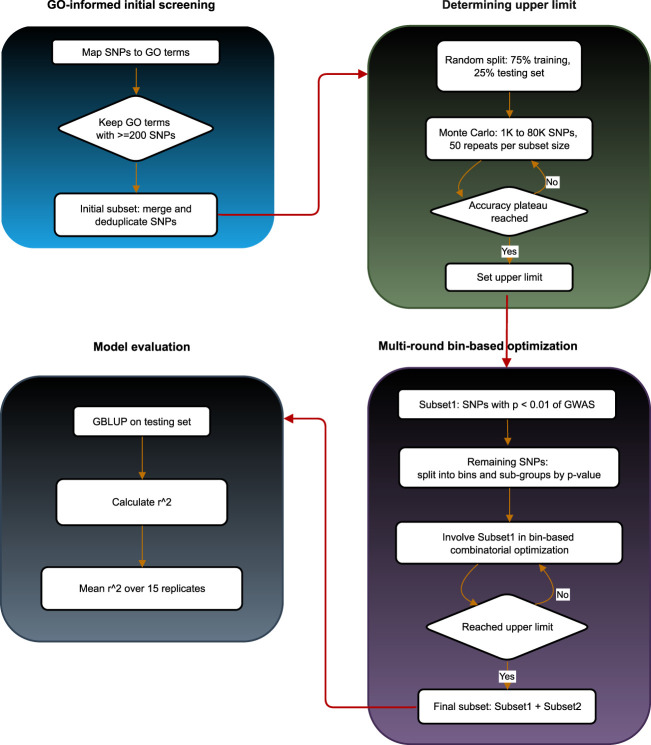
Overall workflow of the binGO-GS pipeline.

To provide a clear overview of the proposed approach, we designed a schematic workflow of the entire binGO-GS pipeline (Figure 3). This diagram summarizes the key steps, including GO-based marker screening, determination of the upper limit for marker subset size, bin-based combinatorial optimization, and model evaluation, offering a comprehensive visualization of the analytical process.

### 2.4 Computational complexity and runtime

The combinational optimization within bins involves selecting a subset of markers within each bin based on performance in cross-validation. Let B be the number of bins (set as 10 in this study), G the number of groups per bin (10 in this study), and F the number of cross-validation folds. The total computational cost is approximately O(B × G × F).

In our implementation, the optimization was run on a workstation with 2.3 GHz Intel Xeon Platinum 9242 CPU (96 cores) and 376 GB RAM. Each trait required about 10 min of processing time with peak memory usage under 32 GB.

### 2.5 Reference models and evaluation strategy

To evaluate the effectiveness of the proposed binGO-GS method–which leverages GO biological priors and bin-based combinatorial optimization for SNP marker subset selection–we conducted a comprehensive comparison with several classical genomic prediction models. The benchmark models include five parametric approaches: GBLUP, Bayes A/B/C, and Bayesian LASSO; a semiparametric method: reproducing kernel Hilbert space (RKHS) regression; and a deep learning model: DNNGP. Among them, GBLUP is also adopted as the base prediction model within the binGO-GS framework.

Model implementations were carried out using either R or Python. GBLUP was implemented via the R package “rrBLUP”; Bayes A/B/C, Bayesian LASSO, and RKHS were implemented using the R package “BGLR”; and DNNGP was implemented with the original DNNGP model (Python-based). For each trait, we randomly portioned the population into training (75%) and testing (25%) sets across 15 independent replicates. SNP marker selection was performed exclusively within the training set, and phenotype of the testing individuals were predicted using the selected markers. To ensure fair comparison, all models used the same data partitions in each replicate. Predictive performance was assessed using the mean coefficient of determination (*r* ^2^) between observed and predicted phenotypic values in the testing set across the 15 replicates. To evaluate the contribution of the final subset to genomic prediction across different traits and models, we compared its performance with that of the full marker set and a randomly selected subset of the same size. Given the hypothesis that the final subset would outperform either baseline, one-tailed paired *t*-tests were conducted to assess the significance of the differences.

## 3 Results and discussion

### 3.1 GO-based biological priors exhibit potential in genomic prediction

The genomic prediction method proposed in this study, binGO-GS, leverages GO-based biological prior knowledge to substantially reduce the number of SNP markers used in prediction. Specifically, only approximately 35% of the full marker set was retained, *i.e.*, 716,860 out of 2,053,821 SNPs for the Arabi944 dataset and 660,238 out of 1,882,667 SNPs for the Arabi407 datasets. Despite this reduction, the resulting SNP sets are still large (∼700 K), posing challenges for both computational efficiency and model interpretability. To assess the predictive utility of biological priors derived from GO, we first evaluated the genomic prediction accuracy based on SNPs associated with each of the top 10 GO terms (ranked by SNP count) (Table 2). Although the SNPs counts for these GO terms were comparable, prediction accuracies varied widely across traits, highlighting the functional diversity of these GO categories. When using only the SNPs linked to a single GO term, the GP accuracy declined by 5.1%–12.7% compared to using the full marker set, suggesting that individual GO terms alone are insufficient to support effective genomic prediction. We then aggregated SNPs across all effective GO terms (∼700 K markers stated above) and found that the average prediction accuracy improved by 2.5% across traits compared to using all SNPs. This result demonstrates the potential of integrating rich GO-based biological priors and reinforces the importance of marker subset selection in improving genomic prediction.

**TABLE 2:** GP accuracies using SNPs associated with the top 10 or all effective GO terms.

Marker set	No.^b^	CL	RL	DTF2	DTF3	DTF1	DM	SE	GR	FN	Trend (%)^c^
All SNPs	∼2 M/∼1.8M	0.4284	0.5550	0.6750	0.6686	0.6667	0.3849	0.4512	0.3616	0.2902	
GO:0097367^a^	36,174	0.3648	0.4547	0.5800	0.5618	0.5743	0.3693	0.4150	0.3326	0.2614	−12.7
GO:0032553	35,219	0.3609	0.4516	0.5805	0.5606	0.5743	0.3757	0.4215	0.3358	0.2646	−12.4
GO:0017076	34,492	0.3598	0.4522	0.5803	0.5597	0.5719	0.3783	0.4263	0.3391	0.2640	−12.3
GO:0032555	34,302	0.3604	0.4522	0.5802	0.5594	0.5726	0.3761	0.4243	0.3369	0.2638	−12.4
GO:0030554	31,663	0.3589	0.4501	0.5802	0.5584	0.5707	0.3757	0.4215	0.3363	0.2566	−12.8
GO:0032559	31,513	0.3594	0.4505	0.5802	0.5584	0.5714	0.3734	0.4196	0.3343	0.2563	−12.9
GO:0009620	29,930	0.3929	0.5225	0.6418	0.6170	0.6299	0.3312	0.4215	0.3155	0.2665	−7.6
GO:0030054	29,304	0.3857	0.5126	0.6518	0.6409	0.6465	0.3764	0.4172	0.3496	0.2729	−5.1
GO:0005911	29,292	0.3857	0.5128	0.6519	0.6408	0.6465	0.3768	0.4168	0.3497	0.2731	−5.1
GO:0080134	28,331	0.3915	0.5014	0.6252	0.6155	0.6202	0.3633	0.4213	0.3347	0.2758	−7.4
Effec. GO	∼717 K/∼660K	0.4400	0.5740	0.6843	0.6792	0.6771	0.4034	0.4604	0.3740	0.3015	2.5

^a^ GO, terms containing the largest number of markers.

^b^ Number of SNPs. c: Average percentage change in prediction accuracy across traits relative to using all SNPs (negative values indicate a decline; positive ones indicate an improvement).

### 3.2 Genomic prediction with different models

**FIGURE 4:**
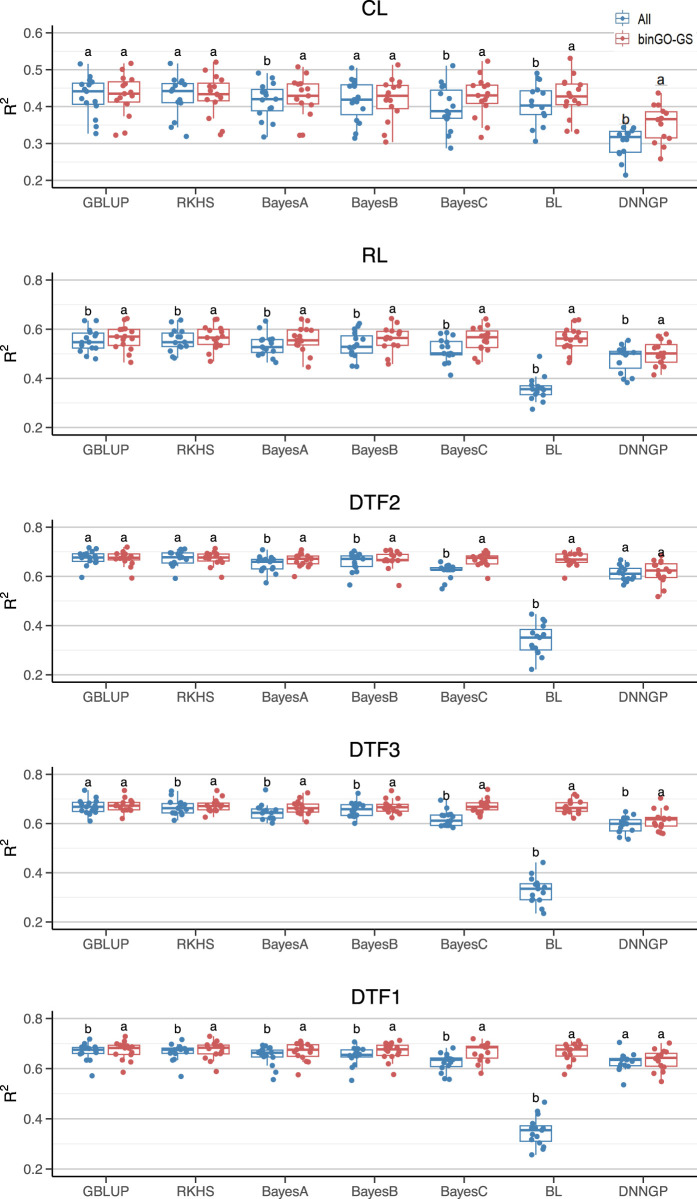
Comparison of GP performance using different models based on the full SNP set and the final subset in the Arabi944 dataset. Boxplots represent prediction accuracies across 15 replicates for each model. Different letters indicate statistically significant differences (*p* < 0.05) between the two marker sets.

**FIGURE 5:**
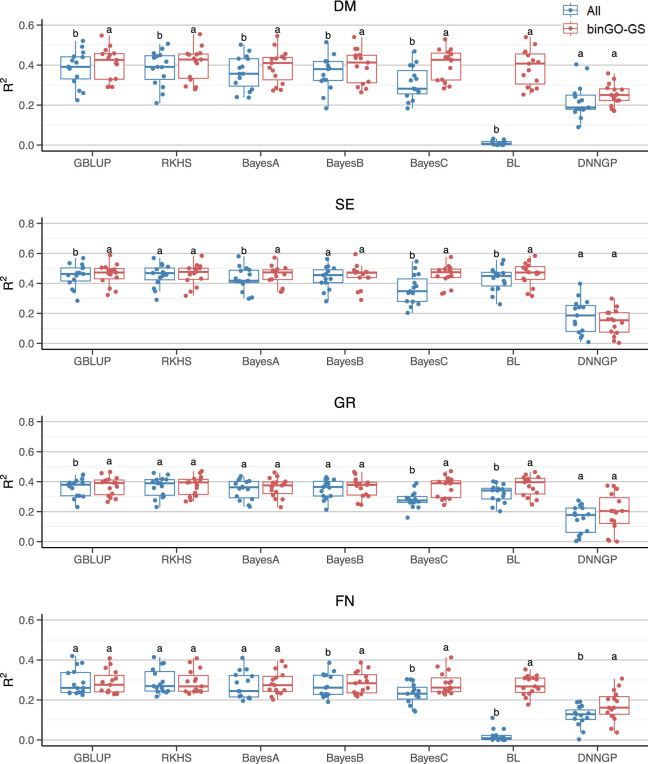
Comparison of GP performance using different models based on the full SNP set and the final subset in the Arabi407 dataset. Boxplots represent prediction accuracies across 15 replicates for each model. Different letters indicate statistically significant differences (*p* < 0.05) between the two marker sets.

Starting from the GO-informed initial marker subset, the final subset was derived through the bin-based combinatorial optimization procedure embedded in binGO-GS. Compared with the full marker set, the final subset significantly improved genomic prediction accuracy across nine traits using the GBLUP model (*p* = 0.0134; Table 3). An effective SNP marker subset should consistently enhance prediction performance across various models. To further validate the robustness and generalizability of binGO-GS, we compared the predictive performance of the selected final subset with that of the full marker set and Subset I (representing strong-effect markers) across nine traits using six additional reference models (Table 3; Figures 4, 5).

**TABLE 3:** Genomic prediction performance for nine quantitative traits in *A. thaliana* using different models.

Models	CL	RL	DTF2	DTF3	DTF1	DM	SE	GR	FN	*p*-value
GBLUP	0.4284^a^	0.5550	0.6750	0.6686	0.6667	0.3849	0.4512	0.3616	0.2902	0.0134^f^
	0.3827^b^	0.5243	0.6273	0.6298	0.6167	0.3934	0.4415	0.3525	0.2713	^g^1.77 × 10^−4^
	0.4308^c^	0.5652	0.6736	0.6729	0.6725	0.4061	0.4590	0.3692	0.2885	
RKHS	0.4267	0.5552	0.6733	0.6656	0.6667	0.3845	0.4530	0.3652	0.2939	0.0086
	0.3842	0.5258	0.6323	0.6335	0.6238	0.3945	0.4434	0.3503	0.2723	9.19 × 10^−5^
	0.4314	0.5668	0.6729	0.6728	0.6742	0.4059	0.4601	0.3723	0.2907	
BayesA	0.4156	0.5353	0.6484	0.6474	0.6511	0.3627	0.4312	0.3482	0.2730	2.35 × 10^−5^
	0.3818	0.5215	0.6273	0.6290	0.6202	0.3951	0.4415	0.3481	0.2676	7.19 × 10^−4^
	0.4259	0.5589	0.6668	0.6647	0.6666	0.3934	0.4550	0.3611	0.2834	
BayesB	0.4161	0.5371	0.6581	0.6567	0.6506	0.3613	0.4388	0.3524	0.2727	6.45 × 10^−4^
	0.3836	0.5209	0.6192	0.6244	0.6140	0.3903	0.4397	0.3477	0.2698	8.33 × 10^−4^
	0.4196	0.5613	0.6682	0.6693	0.6688	0.3947	0.4484	0.3614	0.2848	
BayesC	0.3976	0.5155	0.6211	0.6195	0.6243	0.3094	0.3606	0.2808	0.2310	4.02 × 10^−5^
	0.3835	0.5252	0.6244	0.6271	0.6195	0.3946	0.4417	0.3474	0.2720	2.7 × 10^−4^
	0.4273	0.5583	0.6676	0.6716	0.6654	0.4039	0.4551	0.3643	0.2850	
BL	0.4082	0.3573	0.3429	0.3288	0.3500	0.0107	0.4295	0.3206	0.0213	0.0011
	0.3816	0.5251	0.6279	0.6290	0.6241	0.3926	0.4428	0.3499	0.2742	0.0016
	0.4259	0.5603	0.6700	0.6670	0.6657	0.3875	0.4618	0.3716	0.2730	
DNNGP	0.2960	0.4772	0.6108	0.5943	0.6268	0.2209	0.1789	0.1498	0.1202	0.0236
	0.3154	0.5351	0.6214	0.6253	0.6328	0.2625	0.0773	0.0641	0.1650	0.1394
	0.3413	0.5020	0.6183	0.6135	0.6349	0.2544	0.1401	0.1930	0.1728	
*p*-value	0.0185^d^	0.0557	0.1226	0.1056	0.11	0.063	0.1226	0.0168	0.0837	
	^e^4.24× 10^–6^	0.0172	8.01× 10^–4^	0.0024	4.53× 10^–4^	0.1695	0.0105	0.0413	0.0016	

Note: GP, accuracies were obtained using (a) all SNP, markers, (b) Subset I, and (c) the final marker subset selected by binGO-GS., Significant differences across seven models between the final subset and the full marker set (d) (or Subset I (e)) for each trait are indicated in the last two rows. Significant differences across nine traits between the final subset and the full marker set (f) (or Subset I (g)) for each model are shown in the last column.

#### 3.2.1 Improvement in GP accuracy across different models for each trait

The models presenting significant improvements varied across traits (Figure 4). For the CL trait in the Arabi944 dataset (with a heritability of 0.6715), significant improvements (*p* < 0.05) in prediction accuracy were observed using the Bayes A/C, Bayesian LASSO, and DNNGP models. For the RL trait (*h* ^2^ = 0.7774), all seven models demonstrated significant improvements. For the DTF2 trait (*h* ^2^ = 0.8915), significant gains were seen with Bayes A/B/C and Bayesian LASSO. For the DTF3 trait (*h* ^2^ = 0.8544), all models except GBLUP achieved significant improvements. For the DTF1 trait (*h* ^2^ = 0.8812), all models except DNNGP showed significant improvements. The number of models achieving performance gains generally increased with trait heritability, suggesting that higher-heritability traits are more likely to benefit from the proposed marker selection strategy. However, when considering the overall prediction accuracy across models for each trait, only the CL trait showed a significant improvement (Table 3). This discrepancy may be attributed to the relatively low prediction accuracy of the Bayesian LASSO model for the other four traits when using all SNPs (Table 3; Figure 4), resulting in high variability and reduced statistical power in the significance tests. Despite this, the average percentage improvement in prediction accuracy per trait provides a complementary perspective: CL (4.07%), RL (9.63%), DTF2 (9.64%), DTF3 (10.78%), and DTF1 (9.72%). These results highlight a clear trend of performance enhancement, even when statistical significance is not always reached.

For the Arabi407 dataset, all models except DNNGP exhibited significant improvements in GP accuracy for the DM trait (*h* ^2^ = 0.7976) when using the final marker subset. For the SE trait (*h* ^2^ = 0.7603), significant improvements were observed with GBLUP, Bayes A/C, and Bayesian LASSO. In the case of the GR trait (*h* ^2^ = 0.7296), GBLUP, Bayes C, and Bayesian LASSO models showed significant gains. For the FN trait (*h* ^2^ = 0.6746), significant improvements were achieved with Bayes B/C, Bayesian LASSO, and notably, DNNGP (Figure 5). Consistent with previous findings, traits with higher heritability generally benefited from a greater number of models exhibiting improved accuracy. Notably, the deep learning model DNNGP showed significant improvement for the FN trait, despite the relatively small sample size. This is particularly interesting given that deep learning models typically require large dataset to achieve competitive performance in most pattern recognition tasks. In this context, the carefully selected feature subset may have facilitated model’s ability to learn complex phenotypic patterns from limited training data.

**FIGURE 6:**
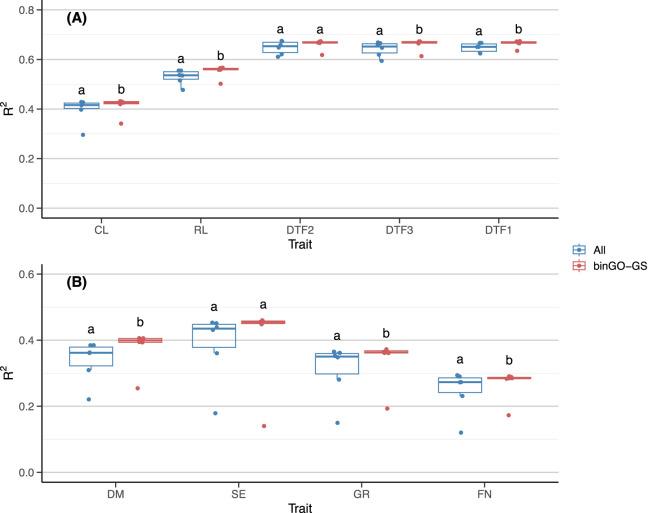
Comparison of GP performance for each trait across different models (excluding Bayesian LASSO) based on the full SNP set and the final subset in **(A)** the Arabi944 and **(B)** the Arabi407 dataset. Each dot in the boxplot represents the mean accuracy of 15 replicates for one model. Different letters above the boxplots indicate statistically significant differences (*p* < 0.05) between the two marker sets.

When evaluating the overall prediction performance across models for each trait, only the GR trait exhibited a significant improvement (Table 3). However, the percentage improvements in accuracy provided a more nuanced view: DM (30.06%) (DM), SE (4.97%), GR (9.84%), and FN (25.02%), highlighting that the DM and FN traits benefited the most in terms of practical performance gains, despite not always reaching statistical significance in formal tests. Interestingly, when the Bayesian LASSO model was excluded from significance tests, three out of four traits (except SE) showed significant improvements, aligning more closely with observed percentage gains (Figure 6B). Similarly, for the Arabi944 dataset, all traits except the DTF2 showed significant improvements after omitting the Bayesian LASSO model (Figure 6A). Overall, the binGO-GS method demonstrated varying yet generally positive effects on genomic prediction accuracy across different traits.

#### 3.2.2 Improvement in GP accuracy across different traits for each model

We then evaluated the contribution of the final subset to each model across multiple traits (Table 3; Figures 4, 5). For the GBLUP model, significant improvements were observed for the RL and DTF1 traits in the Arabi944 dataset, and for all traits except FN in the Arabi407 dataset. For lower-heritability traits such as CL and FN, the performance using the final subset was comparable to that using the full marker set. Overall, the final subset generally enhanced GBLUP performance. For the RKHS model, significant improvements were observed in the RL, DTF3, and DTF1 traits of the Arabi944 dataset, and only the DM trait in the Arabi407 dataset. The limited improvement in the second dataset may be due to the RKHS model’s reliance on a Gaussian kernel, which typically requires larger sample sizes for optimal performance.

For the Bayes series of models, BayesA model showed significant improvement in 7 out of 9 traits (except GR and FN in Arabi407), highlighting the benefit of the final marker subset. This may be attributed to BayesA’s assumption that all markers have effects following a normal distribution with marker-specific variances drawn from a scaled inverse chi-square distribution. Such a flexible yet broad assumption leaves room for performance enhancement through informative marker selection, which can compensate for potential model misfit. BayesB, which assumes that only a small proportion of markers have effects, further aligns with the concept of feature selection. Accordingly, it showed improvements in six traits. BayesC, a computationally simplified version of BayesB that assumes a common effect variance among non-zero markers, initially performed worse than BayesB when using the full marker set–likely due to its restrictive assumption being unsuitable for large, noisy feature sets. However, when applied to the final subset, BayesC achieved significant improvements across all traits, indicating that once non-informative markers are removed, even simpler models can perform well. In contrast, the Bayesian LASSO model performed poorly on 6 of 9 traits when using all markers, likely due to a mismatch between the assumed Laplace distribution of marker effects and the underlying genetic architecture. However, when using the final subset, its prediction performance normalized across all traits. This suggests that inclusion of key causal variants can substantially mitigate model-assumption mismatches, enabling robust prediction even under non-ideal assumptions.

For DNNGP, significant improvements were observed in 4 out 9 traits, with slight increases seen in the others except for SE. However, its overall prediction accuracies, regardless of using the full marker set or the final subset, remained lower than those of other models. This aligns with the known limitation of deep learning models, which typically require larger sample sizes to fully realize their predictive potential.

**FIGURE 7:**
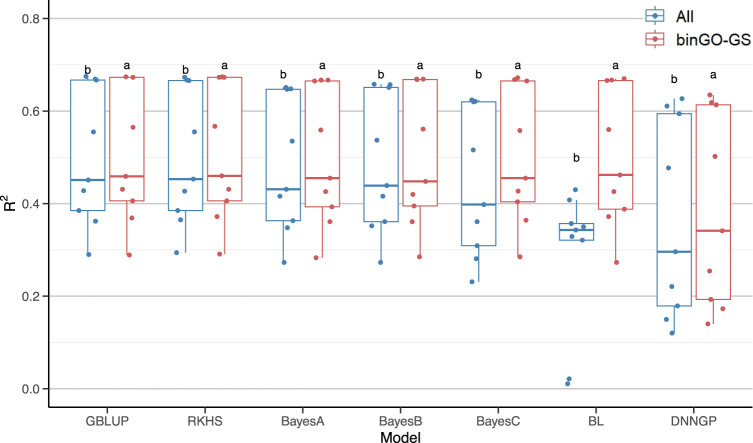
Comparison of GP performance across nine traits for each model using all SNP markers and the final subsets. Each dot in the boxplot represents the mean accuracy of 15 replicates for a single trait. Different letters above the boxplots indicate statistically significant differences (*p* < 0.05) between the two marker sets.

We subsequently assessed whether the use of the optimized marker subsets could enhance the overall prediction performance of each model across multiple traits (Table 3). Despite notable variation in accuracy among traits, all seven models exhibited significant improvements (Figure 7). In summary, while the optimized subsets generated by the binGO-GS algorithm may not improve prediction performance for every individual trait or model, the consistent and often substantial improvements observed across most models and traits underscore the effectiveness and robustness of binGO-GS in genomic selection.

### 3.3 The SNP subset selected by binGO-GS significantly outperforms randomly selected markers

Quantitative traits in *Arabidopsis thaliana* are often polygenic, with each gene exerting a small effect (Zan and Carlborg, 2019; Kearsey et al., 2003). As such, randomly sampling a large number of SNPs across the genome may still yield acceptable prediction accuracy due to the coverage of multiple QTLs through linkage disequilibrium (LD) (Shikha et al., 2017; Li et al., 2018). To verify the effectiveness of marker subsets identified by binGO-GS, we compared them with randomly selected SNP subsets of equal size across 15 replicates for each trait. The final subset contained on average 48,910 ± 7,194 (CL), 41,397 ± 7,057 (RL), 47,419 ± 4,014 (DTF2), 47,667 ± 3,856 (DTF3), and 45,399 ± 2,670 (DTF1) SNPs in the Arabi944 dataset; and 43,858 ± 6,358 (DM), 34,895 ± 5,681 (SE), 40,235 ± 4,685 (GR), and 54,873 ± 10,308 (FN) SNPs in the Arabi407 dataset. In each replicate, training and testing partitions were hold constant, and 500 random subsets were generated. Genomic prediction was performed using GBLUP, and the average accuracy across the random subsets was compared to that of binGO-GS.

**FIGURE 8:**
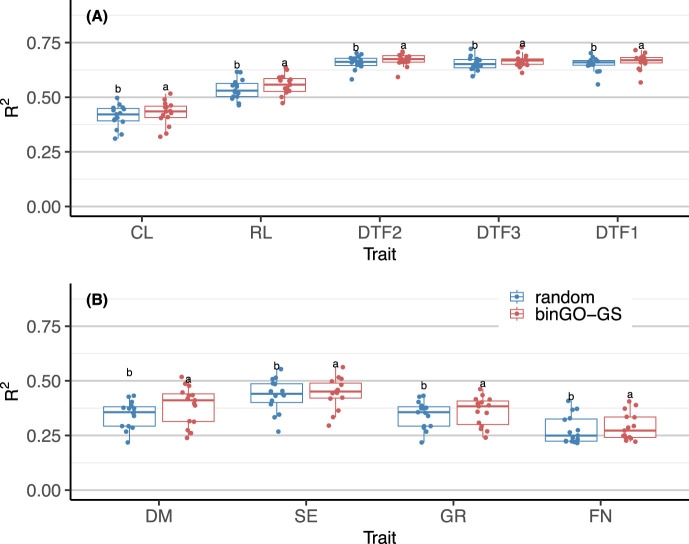
Comparison of GP performance for each trait using GBLUP based on marker subsets selected by binGO-GS and randomly selected markers in **(A)** the Arabi944 and **(B)** the Arabi407 dataset. Each dot in the boxplot represents the mean accuracy across 15 replicates. Different letters above the boxplots indicate statistically significant differences (*p* < 0.05) between the two marker sets.

Results showed that for all traits in both datasets, binGO-GS consistently outperformed random subsets with high statistical significance: for Arabi944 with *p*-values of 1.32 × 10^−6^ (CL), 2.85 × 10^−6^ (RL), 1.49 × 10^−4^ (DTF2), 2.71 × 10^−5^ (DTF3), and 1.38 × 10^−7^ (DTF1) (Figure 8A); for Arabi407 with *p*-values of 6.23 × 10^−5^ (DM), 3.52 × 10^−4^ (SE), 8.08 × 10^−6^ (GR), and 0.003(FN) (Figure 8B). These findings confirm the validity of binGO-GS in identifying informative marker subsets. While randomly selected SNPs can still retain reasonable prediction performance, like due to coverage of QTLs via LD, they lack consistency and interpretability. Moreover, the number of markers needed in such random schemes is difficult to determine in practice. Our previous work has also demonstrated that the inclusion of non-causal variants can significantly impair prediction accuracy, especially in cross-population scenarios (Dai et al., 2020). Additionally, even though a Monte Carlo-based upper limit for marker subset size can be estimated, randomly selected SNPs are less practical for downstream breeding applications or biological interpretation.

### 3.4 Contribution of weak-effect markers in binGO-GS to genomic prediction

As described in the Methods section, once the final marker subset was determined, we defined the marker set excluding the strong-effect markers in Subset I as Subset II, which primarily contains weak-effect markers. To evaluate its contribution, we compared the genomic prediction performance and marker set sizes across subsets. Using the full marker set as a baseline, Subset I alone led to a 5.06% average decrease in GP accuracy across nine traits, despite comprising only 6,808 SNPs on average (Table 3 and 4). This suggests that relying solely on strong-effect markers is insufficient to capture the full genetic architecture of complex traits. In contrast, Subset II achieved a slight but statistically significant improvement in prediction accuracy (*p* = 0.0209), despite excluding the strong-effect markers. The average size of Subset II was 38,287 SNPs, which is substantially smaller than the full set, indicating that genome-wide weak-effect markers still carry sufficient information to capture LD with potential causal variants (Table 4). Combining Subset I and II into the final subset led to further improvements in prediction accuracy (*p* = 0.0134), with an average of 44,961 SNPs (Table 4). This highlights the necessity of integrating both strong- and weak-effect markers to enhance prediction by capturing complementary LD signals related to true quantitative trait nucleotides (QTNs).

**TABLE 4:** Genomic prediction accuracy and marker set sizes for different subsets involved in binGO-GS.

Dataset	Trait	Subset I	Subset II	Final subset
Arabi944	CL	7,063 ± 289^a^	41,990 ± 5,681	48,910 ± 7,194
		0.3827 ± 0.0562^b^	0.4307 ± 0.0404	0.4308 ± 0.0582
	RL	6,306 ± 80	35,180 ± 6,026	41,397 ± 7,057
		0.5243 ± 0.0535	0.562 ± 0.0375	0.5652 ± 0.0519
	DTF2	6,938 ± 207	40,863 ± 3,587	47,419 ± 4,014
		0.6273 ± 0.0312	0.6732 ± 0.0204	0.6736 ± 0.0304
	DTF3	6,862 ± 154	40,927 ± 3,436	47,667 ± 3,856
		0.6298 ± 0.033	0.6709 ± 0.0194	0.6729 ± 0.0279
	DTF1	6,762 ± 157	38,753 ± 2,245	45,399 ± 2,670
		0.6167 ± 0.0370	0.6729 ± 0.0285	0.6725 ± 0.0369
Arabi407	DM	8,595 ± 151	35,363 ± 4,687	43,858 ± 6,358
		0.3934 ± 0.0831	0.3988 ± 0.0682	0.4061 ± 0.0834
	SE	6,632 ± 232	28,340 ± 6,193	34,895 ± 5,681
		0.4415 ± 0.0688	0.4589 ± 0.0566	0.459 ± 0.0733
	GR	6,695 ± 211	33,611 ± 3,977	40,235 ± 4,685
		0.3525 ± 0.0691	0.3697 ± 0.0545	0.3692 ± 0.0652
	FN	5,417 ± 79	49,555 ± 8,113	54,873 ± 10,308
		0.2713 ± 0.0561	0.2855 ± 0.0528	0.2885 ± 0.0614

^a^ Average number of markers (with standard deviations) across 15 replicates

^b^ Average prediction accuracy (with standard deviations) across15 replicates.

Trait-level comparisons revealed that Subset II improved prediction accuracy over Subset I for all nine traits, with increases ranging from 6.53% to 12.54% in Arabi944% and 1.37%–5.23% in Arabi407. Moreover, the final subset outperformed Subset II for seven of the nine traits (Table 4), though with modest gains. Importantly, Subsets I and II had no overlapping markers for any trait, indicating that they capture distinct yet complementary genetic signals. These findings suggest that both strongly and weakly associated markers contribute synergistically to genomic prediction by jointly modeling diverse genetic effects. Overall, the final subset effectively integrates these complementary signals, resulting in enhanced prediction accuracy.

### 3.5 Distribution of marker subsets across the genome

The genomic distribution of SNP markers associated with different traits in *Arabidopsis thaliana* can vary considerably. We analyzed the preferred distribution patterns of selected SNPs from two perspectives: (1) their distribution across different effect-size intervals (based on *p*-values), and (2) their genomic locations in relation to gene-coding/noncoding regions. Because the GP accuracy of binGO-GS was evaluated based on the average of 15 replicates, each trait resulted in 15 sets of marker subsets. Taking the intersection of these sets may omit informative SNPs, whereas taking the union may introduce non-representation markers. Therefore, to explore the genomic distribution characteristics, binGO-GS was applied once per trait using the full sample set to generate a representative marker subset. For the five quantitative traits in the Arabi944 dataset, the number of selected SNPs ranged from 41,296 to 51,700, corresponding to 2.01%–2.52% of all markers. For the four traits in the Arabi407 dataset, the number ranged from 37,399 to 62,025, representing 1.99%–3.29% of total markers.

**FIGURE 9:**
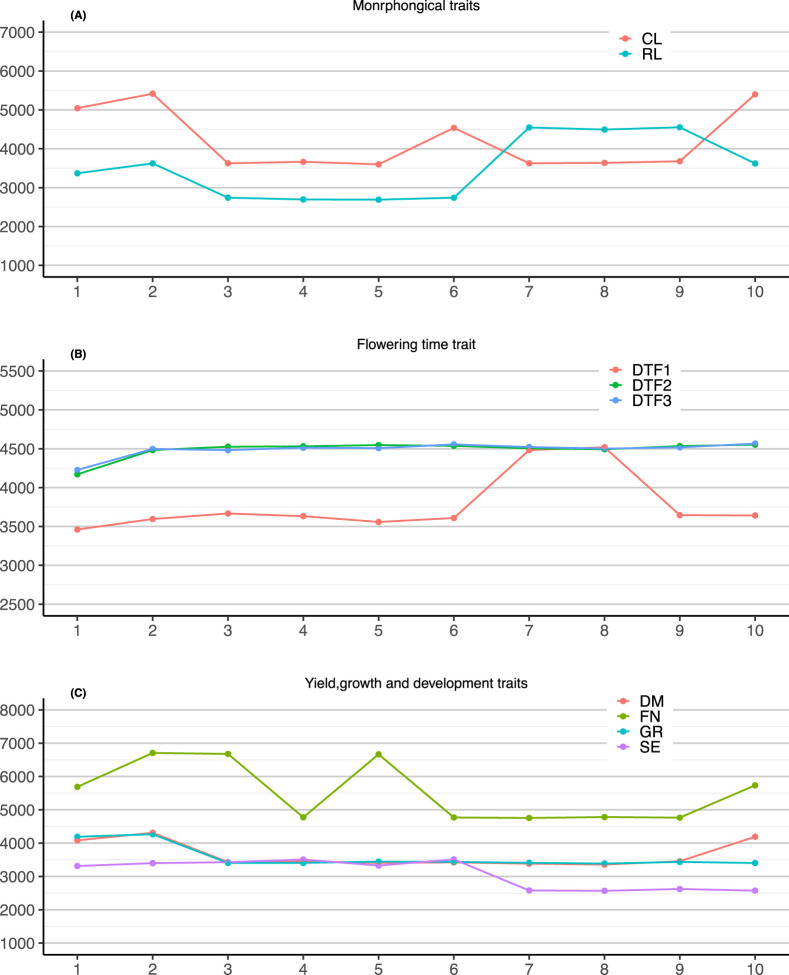
**(A)** Morphological traits, including CL and RL; **(B)** Flowering time traits, including DTF2, DTF3, and DTF1; **(C)** Yield (DM), growth-related traits (SE and GR), and fruit growth and development trait (FN).

We first examined the distribution of selected markers across effect-size intervals (*p*-value intervals: 0.01–0.1, 0.1–0.2…, 0.9–1). Markers with *p*-value <0.01 showed relatively consistent numbers across traits and were thus excluded from comparative analysis. Interestingly, although both CL (stem branching number) and RL (rosette leaf number) are morphological traits, their marker distributions differed (Figure 9A). This is likely due to their anatomical differences, with CL being a stem-coordinate trait and RL an organ-structural trait. The three flowering time traits (DTF2, DTF3, DTF1) exhibited similar distribution patterns, with particularly close resemblance between DTF2 and DTF3 (Figure 9B). Traits such as DM (yield-related) and GR (growth-related) belong to different functional categories but both related to plant dry weight, which may explain their similar marker distribution. In contrast, the FN (fruit growth and development) showed a more distinct distribution pattern (Figure 9C).

Overall, traits within the same biological category (e.g., DTF2 and DTF1) tended to have similar distributions across *p*-value intervals, reflecting shared genetic architectures. Even among traits within the same category but differing in anatomical or developmental context (e.g., SE, GR, FN), subtle differences in marker distribution were observed. These results suggest that the consistency of distribution patterns across effect-size intervals can provide insight into the genetic relatedness of traits, and further serve as an auxiliary strategy to evaluate the biological validity of marker subset selection methods.

**FIGURE 10:**
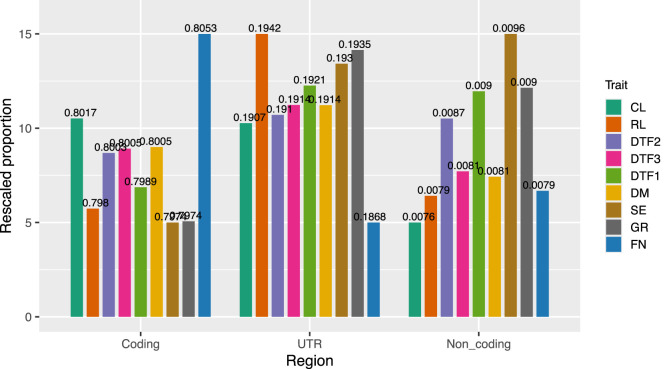
Distribution of the final marker subset across genomic regions, including coding, noncoding, and untranslated regions (UTR). Noncoding regions include various noncoding RNAs such as lncRNAs, rRNAs, and tRNAs. For each trait, the proportion of markers in each region was scaled to a common range (5–15) to allow cross-trait comparison. The original proportion was marked above each bar.

Further analysis was performed to investigate the distribution of the final marker subsets across coding regions, noncoding regions, and untranslated regions (UTRs) in the genome. Noncoding regions were defined to include various types of noncoding RNAs, such as lncRNAs, rRNAs, and tRNAs, while UTRs were considered separately. To ensure comparability of marker numbers across different traits within each genomic region, the proportion of markers for each trait in a specific region (i.e., the proportion of markers in the coding region relative to the total number across all regions) was scaled to a range of 5–15 (Figure 10).

For the CL trait, the rank of the proportions of markers in the coding and UTR regions were similar and substantially higher than that in the noncoding region. In contrast, for the RL trait, the rank of proportion in the UTR region dominated over the other two, consistent with the findings from the effect-size interval comparison between these two traits. For the developmental traits DTF2 and DTF1, the distribution of SNP markers across the genomic regions was generally similar, with comparable proportions in the UTR and noncoding regions. DTF3 and DM exhibited highly consistent marker proportions across all three regions. Although DM is a yield-related trait (rosette mass) and DTF3 is related to flowering time, the similarity in marker distribution may suggest a potential association between rosette biomass and flowering time. The traits SE and GR, both related to stem growth and development, showed similar overall distribution patterns, although the proportions in the UTR and noncoding regions differed. FN, a trait related to fruit growth and development, exhibited a more distinct distribution, with a higher proportion of markers located in coding regions. Notably, the prediction performance for FN was much lower than that of the other traits, which may be attributed to the exclusion of causal variants located in the untranslated regions during marker subset selection.

### 3.6 Practical considerations and opportunities for cost-effective genotyping using binGO-GS

Although binGO-GS provides a theoretically efficient subset of informative SNPs, its practical implementation in breeding programs faces technical and economic constraints. Most existing genotyping platforms, such as fixed-content SNP arrays (e.g., Illumina chips), are not designed to accommodate small, customized SNP panels (Rasheed et al., 2017). Even when only a few hundred SNPs are selected by binGO-GS, breeders are required to pay for the entire chip. Targeted sequencing offers flexibility for SNP selection but is limited by high setup costs for small-scale use, including probe design and validation (Thomson, 2014).

Nonetheless, in scenarios where custom genotyping is technically feasible, such as in high-value breeding targets (e.g., hybrid rice parental lines or dairy bulls) or research-driven programs, binGO-GS-selected panels can offer cost advantages. For such populations, which require repeated genotyping over time, the marginal cost of applying a low-density, binGO-GS-guided panel (e.g., via targeted sequencing) can be lower than full-genome sequencing or fixed chips. For users limited to commercial arrays, a practical compromise is to intersect binGO-GS SNPs with existing chip content and use overlapping markers, avoiding additional genotyping costs while retaining part of the method’s predictive advantage.

With the advancement of flexible genotyping platforms, such as liquid-phase SNP chips (e.g., Axiom) and cost-efficient multiplex PCR systems (e.g., GT-seq), the technical barriers to custom SNP panel deployment are gradually decreasing (Campbell et al., 2015). In the future, binGO-GS may be integrated with genotype imputation approaches, allowing low-density panels to recover high-resolution genotypes by referencing dense panels. Such strategies can further enhance prediction accuracy without increasing genotyping costs, making binGO-GS a forward-looking approach with practical potential for low-cost, high-efficiency genomic selection.

### 3.7 Characteristics and limitations of binGO-GS

The GO-informed selection of marker subset was inspired by a previous study (Farooq et al., 2021). They incorporated gene ontology information into genomic prediction by modeling each GO term as a separate genomic component in the GFBLUP framework. Specifically, markers associated with a given GO term were used to construct a genomic relationship matrix (Gf), and the remaining markers formed a second matrix (Gr), allowing partitioning of the total genomic value. The predictive power of each GO term was then assessed across 7,297 terms using repeated 8-fold cross-validation, resulting in over 583,000 models per trait. While effective in identifying informative GO terms, this exhaustive modeling is computationally intensive and impractical for large-scale breeding programs. Moreover, relying solely on the top-performing GO term may miss relevant genetic signals, while combining all associated GO terms risks redundancy and diminished accuracy.

In contrast, binGO-GS integrates GO knowledge prior to model training. It first aggregates markers from GO terms that meet a minimum size threshold (e.g., ≥200 markers) to retain robust biological context. These markers are then subjected to supervised selection through combinatorial optimization within effect-size–based bins defined by GWAS *p*-values. This design leverages both biological priors and trait association strength, while avoiding model proliferation and redundancy issues observed in previous studies.

Despite the promising results obtained on two Arabidopsis datasets covering nine traits, the generalizability of the proposed method to other species remains to be further explored. Arabidopsis was chosen primarily due to its status as a model plant with abundant high-quality genomic and phenotypic data. Crops such as maize and rice differ in genome complexity, LD patterns, and marker density, which may affect the performance of biologically informed feature selection methods. Additionally, some traits in the Arabi407 dataset, such as FN with a sample size of 396, involve relatively small populations, which may raise concerns about model stability. To mitigate this issue, we employed repeated experiments and ensured that the model performance is not dominated by data partitioning variance. Nonetheless, small sample sizes inherently limit statistical power and might restrict the model’s ability to generalize. This constraint should be carefully considered when interpreting the results. Future work will apply binGO-GS to additional species and explore the integration of crop-specific GO annotations. Moreover, simulation-based evaluations could serve as an intermediate step to assess the method’s robustness across varying genomic architectures.

## 4 Conclusion

In this study, we proposed a biologically informed SNP selection method, binGO-GS, to enhance genomic prediction for nine quantitative traits across two Arabidopsis datasets. By integrating gene ontology knowledge with marker selection, binGO-GS effectively identifies trait-informative SNP subsets, enabling improved prediction accuracy, minimized marker redundancy, and potentially reduced genotyping costs. Across all nine traits, binGO-GS significantly outperformed random marker selection in terms of predictive performance. Moreover, when compared to using the full marker set, binGO-GS consistently achieved higher prediction accuracy across seven statistical models. These results demonstrate the method’s robustness and generalizability. binGO-GS provides a practical framework for designing low-density genotyping panels while maintaining high prediction accuracy. It also offers a valuable tool for dissecting the genetic architecture of complex traits, contributing to more efficient genomic selection and accelerating progress in crop improvement and germplasm innovation.

## Data Availability

The original contributions presented in the study are included in the article/supplementary material, further inquiries can be directed to the corresponding author.
